# *Saprochaete capitata* Infection in Teen With Acute Myeloid Leukemia Receiving Echinocandin Prophylaxis

**DOI:** 10.1155/crdi/4459447

**Published:** 2024-11-20

**Authors:** Jordan Killingsworth, Rachel Boren, Rachna Sheth, Michael L. Chang

**Affiliations:** ^1^Department of Internal Medicine-Pediatrics, McGovern Medical School at UTHealth Houston, Houston, Texas, USA; ^2^Department of Pediatrics, McGovern Medical School at UTHealth Houston, Houston, Texas, USA; ^3^Department of Pediatrics, Division of Pediatric Hospital Medicine, The University of Texas MD Anderson Cancer Center, Houston, Texas, USA

**Keywords:** AML, echinocandin resistance, *Saprochaete capitata*

## Abstract

We report a case of a 15-year-old with refractory and relapsed AML and profound prolonged neutropenia who developed a *Saprochaete capitata* disseminated invasive infection while on echinocandin prophylaxis for invasive fungal disease. Azole antifungal therapies, which are often used as prophylaxis, were initially avoided due to concerns for CYP drug interactions. Treatment with a combination of liposomal amphotericin B, voriconazole, and adjuvant granulocyte transfusions was successful as he awaited neutrophil recovery.

## 1. Introduction

Opportunistic fungal infections are increasing among those with hematologic malignancies and those receiving chemotherapy due to generally immunocompromised state, neutropenia, changes in hematopoietic stem cell transplantation practices, and development of new chemotherapy agents and regimens with unique infectious complications [1–3]. Acute myeloid leukemia (AML) patients are often at increased risk of invasive fungal disease (IFD) compared to acute lymphoid leukemia patients due to increased intensity of chemotherapy and prolonged duration of neutropenia, and IFD remains a significant cause of morbidity and mortality for AML patients [4–6]. Pediatric patients with AML are recommended to receive systemic antifungal prophylaxis with a mold-active agent, which include conventional and lipid formulations of amphotericin, echinocandin, or mold-active azoles [1]. *Candida* and *Aspergillus* remain among the most common fungal infections in AML patients [7, 8], and reducing proven or probable IFD appears comparable between echinocandins and mold-active azoles [1, 9].

Azoles act to inhibit fungal cytochrome P450_14DM_, making triazoles potent inhibitors of CYP enzyme systems leading to significant drug interactions [10]. Consequently, mold-active azoles are more likely to be associated with discontinuation of antifungal prophylaxis due to an adverse effect [1, 9]. As such, echinocandins are often used as prophylaxis against IFD in patients with hematologic malignancies [11], and trials of new chemotherapeutic agents for pediatric leukemia patients may avoid the use of azoles due to their effect on CYP enzymes.

Breakthrough infections with resistant organisms while receiving echinocandin prophylaxis remain a challenge. According to the Global Guideline for the Diagnosis and Management of Rare Yeast, echinocandin monotherapy for prophylaxis can put patients at risk for *Saprochaete capitata* infection [12], a yeast that appears intrinsically resistant to echinocandins.

We report a case of systemic infection due to *Saprochaete capitata* in an immunocompromised teenager receiving echinocandin IFD prophylaxis.

## 2. Case Presentation

A 15-year-old male with relapsed, refractory AML status post matched sibling hematopoietic stem cell transplant receiving chemotherapy that precluded mold-active azole IFD prophylaxis was admitted for worsening neuropathic pain, anorexia, decreased PO tolerance, and febrile neutropenia. At this time it was determined that the patient's AML was refractory to current therapy and the patient was transitioned to cladribine, idarubicin, cytarabine, gemtuzumab, and venetoclax, a regimen more immunosuppressive beyond the typical induction chemotherapy for relapsed/refractory pediatric AML [13]. For febrile neutropenia, blood cultures were obtained, and the patient was initiated on vancomycin and cefepime. Caspofungin (50 mg IV once daily every 24 h) for IFD prophylaxis was continued. The patient remained febrile with all other vital signs normal. The patient also had persistent complaints of esophageal and chest pain with swallowing and physical findings of mucositis and pharyngeal white exudate that only minimally responded to glutamine mouth washes, pantoprazole, carafate, and 2% xylocaine medications. The patient continued to be febrile despite broad-spectrum antibiotic therapy of vancomycin and cefepime for two weeks with 10 negative blood cultures during this time period. Vancomycin was continued and cefepime was transitioned to meropenem. The patient developed hypotension on hospital day 17, with concerns of septic shock resistant to fluid resuscitation. The patient was transferred to the pediatric intensive care unit (PICU) for vasopressor therapy. Subsequently on hospital day 18 a blood culture obtained on hospital day 15 from the white lumen of a 5 Fr power rated antibiotic coated left basilic vein double-lumen peripherally inserted central catheter (PICC) line (Teleflex) identified fungal elements at 3 days 2 h and 17 min. This PICC line had been inserted 61 days prior to the date positive blood culture was obtained. Culture from the orange (second) lumen of the PICC line obtained at the same time was negative. Repeat blood cultures obtained on hospital day 15 from both lumens of the PICC line 18 h after the first set also became positive, with the white lumen becoming positive for fungal elements at 1 day 2 h and 50 min, and the orange lumen positive at 3 days 16 h and 10 min. BioFire BCID Panel was negative for identification of *Candida* spp. and *Cryptococcus* spp.

With identification of fungal elements despite caspofungin therapy, voriconazole therapy (6 mg/kg IV loading dose) was initiated for fungal sepsis while caspofungin was discontinued. As the patient was critically ill with history of prolonged neutropenia (7 weeks by this time), liposomal amphotericin B (5 mg/kg/dose, once daily every 24 h) was added in addition to voriconazole (200 mg IV every 12 h). Complicating matters, this patient had a recorded confirmed anaphylactic-type reaction (rash and shortness of breath) to amphotericin B previously and underwent amphotericin B desensitization in the PICU (described in discussion). The identity of the yeast was confirmed as *Saprochaete capitata* approximately 48 h after initial isolation from blood cultures and was sent to a reference laboratory for susceptibility testing. An echocardiogram was performed that showed no evidence of vegetation. Computed tomography (CT) imaging of the chest, abdomen, and pelvis without contrast demonstrated new “multifocal lung opacities and small left pleural effusion” compatible with new infectious pneumonia. Blood cultures obtained on hospital day 17 and early morning hospital days 18 from the PICC line were also positive for *Saprochaete capitata*. The left basilic vein PICC line was removed and a new 5 Fr power rated antibiotic coated double lumen PICC line was placed in the right brachial vein on hospital day 18. One additional blood culture obtained from the new right brachial PICC line and approximately 8 h after left brachial PICC line was removed was positive for *S. capitata* (hospital day 19, 24 h after initiation of liposomal amphotericin B, 41 h after first dose of voriconazole). PICC catheter tip culture from the removed left basilic PICC was negative. Following left basilic PICC removal, subsequent blood cultures obtained starting hospital day 20, 5 days after the first positive culture all remained negative. Susceptibility testing of the *S. capitata* isolate revealed amphotericin B minimum inhibitory concentration (MIC) of 1 mcg/mL and voriconazole MIC ≤ 0.03 mcg/mL.

The patient was also started on colony-stimulating factor filgrastim-sndz 10 mcg/kg intravenously (Zarxio) the same day that the first blood culture was reported to be positive (hospital day 18) for 4 doses. Chemotherapy was held throughout the remainder of the hospital course after the initial induction dose.

After multidisciplinary discussions with the patient's family regarding critically ill status with significant immunosuppression, granulocyte transfusions harvested from family members were utilized as an adjuvant to the combination antifungal regimen and PICC line removal. The patient received a total of 4 granulocyte transfusions on day 2, 3, 6, and 7 (hospital days 20, 21, 24, and 25, respectively, volumes 373 mL, 271 mL, Unit A 272 mL, B 195 mL, A 272 mL, B 195 mL, cell counts not available) after the first blood culture was reported to be positive. Prior to first granulocyte transfusion and 48 h after first dose of filgrastim-sndz, the patient's white blood cell count (WBC) was 0.1 K/*μ*L. Immediately following completion of first granulocyte transfusion, the patient's WBC count was 1.9 K/*μ*L with absolute neutrophil count (ANC) of 1.81 K/*μ*L. Approximately 8 h after the granulocyte infusion, the patients WBC count was 0.6 K/*μ*L with ANC 0.53 K/*μ*L. The patient had similar response to the second and third granulocyte infusions. Finally, following the fourth granulocyte infusion, the patient's WBC count was 4.7 K/*μ*L and ANC 3.24 K/*μ*L. 17 h after the granulocyte infusion the patient's WBC count had decreased to 1.9 K/*μ*L ANC 0.74 K/*μ*L. Fortunately, by 28 h after the fourth granulocyte infusion (hospital day 26, 8 days after the blood culture was first reported positive for *Saprochaete capitata*, 5 days after the last dose of filgrastim-sndz) the patient's WBC count had increased to 3.0 K/*μ*L ANC 0.69 K/*μ*L.

Blood cultures were negative from hospital day 20, prior to granulocyte infusions but following 3 days of filgrastim-sndz, while the ANC was still 0.1 K/*μ*L. With persistent fever and hypotension, a repeat CT sinus and chest without contrast obtained 12 days (hospital day 30) after the prior image revealed no evidence for acute sinusitis, but with interval increase in ground glass and bilateral pulmonary nodules/nodular opacities compatible with pneumonia. CT abdomen and pelvis with contrast noted splenic enlargement with multiple hypodense splenic lesions, as well as several liver hypodensities consistent with disseminated IFD. The patient's total WBC count at the time of repeat imaging was 6.9 K/*μ*L and ANC was 2.28 K/*μ*L. Repeat echocardiogram did not demonstrate any vegetations. The patient was downgraded from PICU status after 18 days (hospital day 34, 14 days after negative blood culture, 8 days after ANC reached > 0.50 K/*μ*L without granulocyte infusions) and antibiotics were de-escalated. Liposomal amphotericin B and voriconazole combination therapy were continued through discharge after a 42-day hospitalization (26 days after onset of fungal sepsis symptoms). Voriconazole level (Mayo) on hospital day 21 (3 days after initiation, but not a trough level) was 8.4 mcg/mL. The voriconazole dose was adjusted on hospital day 25 (7 days after initiation) with follow up voriconazole level (Mayo) on hospital day 31 of 4.5 mcg/mL. The patient was discharged on oral voriconazole 200 mg PO Q12 and liposomal amphotericin B 5 mg/kg once daily on Monday, Wednesday, and Friday. Voriconazole trough (Mayo) at the time of discharge was 2.0 mcg/mL.

## 3. Discussion

Presented here is a rare pediatric case of systemic infection due to *Saprochaete capitata* in a neutropenic teenager on echinocandin IFD prophylaxis requiring amphotericin B desensitization for definitive antifungal therapy, who also received granulocyte transfusions for management. In existing literature, the majority of cases of *S. capitata* infection are in adults greater than 18 years old.

*Saprochaete capitata* (previous names: *Blastoschizomyces capitatus, Trichosporon capitatum, Geotrichum capitatum,* teleomorph *Magnusiomyces capitatus*) is a nonencapsulated ascomycetous yeast that can cause significant morbidity and mortality in neutropenic patients with approximately 104 cases reported in the largest literature review from 1977 to August 2013 [14]. This fungus is found in various locations such as soil, water, plants, and sometimes dairy products and can often colonize the skin, bronchial tree, and intestinal tract [15]. The source of infection for our patient is not clear, but mucosal barrier injury secondary to esophagitis given the initial clinical symptoms and physical exam findings consistent with pharyngitis and esophagitis, or possibly PICC-associated bloodstream infection are plausible. In this case, modification of antifungal therapy and removal of the PICC line was associated with resolution of fungemia, which occurred after starting filgrastim-sndz, but prior to increase of ANC and prior to the first granulocyte infusion.

The spectrum of disease caused by *Saprochaete* is similar to that of *candida*, though invasive tissue disease and disseminated disease are reported to be more common with *S. capitata* which can rapidly progress to multiorgan failure [15]. Previous reports suggest high mortality rate [16]. Our patient had evidence of widely disseminated disease following fungemia given the imaging findings of the lungs, liver, and spleen. Fortunately, our patient did not have any evidence of endocarditis during this episode.

*Saprochaete* species appear resistant to echinocandins, and breakthrough infections may occur while on echinocandin IFD prophylaxis [14, 16]. Arrieta-Aguirre et al. identified a specific mutation in the *FKS* gene from 12 *S. capitata* isolates resulting in a change to *β*-1, 3-D-glucan synthase reducing echinocandin susceptibility [17]. No therapeutic MIC breakpoints exist nor have specific therapeutic regimens been established. Most experts utilize a combination of amphotericin B and other antifungal drugs such as voriconazole [15]. Signs and symptoms of any breakthrough infection while on broad-spectrum antibiotic therapy should also prompt consideration of modification of existing antifungal prophylaxis or therapy, especially if on echinocandin monotherapy.

The need for amphotericin B desensitization complicated initial empiric therapy in this patient with documented anaphylactic-type reaction to prior administration of amphotericin B. Prior to this episode the patient had been desensitized to amphotericin B once at another institution for treatment, so it was expected that the patient would tolerate desensitization again. Our institution's liposomal amphotericin B desensitization protocol has each dose infused over 15 min, followed by 15 additional minutes of observation prior to escalating dose. Doses of 0.06 mg, 0.08 mg, 0.01, 0.1, 1, 2, and 5 mg/kg were administered consecutively without incident. Based on the MICs reported for this *S. capitata* isolate (amphotericin B MIC 1 mcg/mL, voriconazole MIC ≤ 0.03 mcg/mL), likely either antifungal would have been effective though again no specified breakpoints exist.

A relatively unique aspect of this patient's treatment regimen was granulocyte transfusions which are typically reserved for those with neutropenia and evidence of severe infection or infection unresponsive to antibiotics [18]. These transfusions provide a temporary boost to the immune system while patients remain neutropenic but are usually not for long-term management given short half-life of the granulocyte components [18]. A Cochrane review published in 2016 concluded that quality of evidence was very low to low per GRADE methodology due to high risk of bias and “outcomes being imprecise”. This systematic review concluded there “may be no difference in all-cause mortality over 30 days between participants receiving granulocyte transfusions and those that did not [19].” One previous case of an adult patient with *Saprochaete clavata* infection successfully treated with combination antifungal therapy (liposomal amphotericin B and voriconazole) along with adjuvant granulocyte transfusions has been published. That report described neutrophils/transfusion ranging from 2.9 to 4.3 × 10^10^ neutrophils per transfusion for 5 transfusions [20]. For our case, the patient received 4 transfusions with the following volumes: 373 mL, 271 mL, Unit A 272 mL, B 195 mL, A 272 mL, B 195 mL (cell counts unavailable), though impact on clinical outcome remains unclear given the multiple simultaneous interventions such as interruption of chemotherapy, colony-stimulating factor administration, and combination antifungal therapy. Similar to the previous case report, our patient tolerated the granulocyte infusions without obvious adverse effects.

This case highlights an uncommon though emerging infection with *Saprochaete capitata*. While no consensus for optimal therapeutic regimens for disseminated *S. capitata* infections with critical presentation exist, source control and initial therapy with combination triazole and amphotericin B were associated with resolution of fungemia for this patient. Withholding further chemotherapy and initiating colony stimulating factor administration with neutrophil recovery contributed to successful control of disseminated *S. capitata* infection. Adjuvant therapy with granulocyte transfusions were tolerated in this case and may play a role in therapy for infections from rare yeast, though more investigation is required. For patients receiving prolonged echinocandin monotherapy, providers should consider modification of antifungal therapy when any concern of breakthrough infection is present to ensure antifungal activity against *S. capitata* and other rare yeasts.

## Data Availability

Data reported in this single patient case report are maintained in the patient's electronic medical record. Release would violate patient confidentiality.
